# Older and wiser? Men’s and women’s accounts of drinking in early mid-life

**DOI:** 10.1111/j.1467-9566.2011.01424.x

**Published:** 2011-10-28

**Authors:** Carol Emslie, Kate Hunt, Antonia Lyons

**Affiliations:** 1 MRC Social and Public Health Sciences UnitGlasgow, UK; 2 School of Psychology, Massey UniversityNew Zealand

**Keywords:** alcohol, gender, lay knowledge, men’s health, women’s health

## Abstract

Most qualitative research on alcohol focuses on younger rather than older adults. To explore older people’s relationship with alcohol, we conducted eight focus groups with 36 men and women aged 35 to 50 years in Scotland, UK. Initially, respondents suggested that older drinkers consume less alcohol, no longer drink to become drunk and are sociable drinkers more interested in the taste than the effects of alcohol. However, as discussions progressed, respondents collectively recounted recent drunken escapades, challenged accounts of moderate drinking, and suggested there was still peer pressure to drink. Some described how their drinking had increased in mid-life but worked hard discursively to emphasise that it was age and stage appropriate (*i.e*. they still met their responsibilities as workers and parents). Women presented themselves as staying in control of their drinking while men described going out with the intention of getting drunk (although still claiming to meet their responsibilities). While women experienced peer pressure to drink, they seemed to have more options for socialising without alcohol than did men. Choosing not to drink alcohol is a behaviour that still requires explanation in early mid-life. Harm reduction strategies should pay more attention to drinking in this age group.

## Introduction

There is increasing concern about harmful alcohol consumption in the UK (Office for National Statistics (ONS) 2011a), particularly within Scotland (Emslie and Mitchell 2009, Leyland *et al*. 2007, The Scottish Government 2008). Policy and media attention has focused on young ‘binge’ drinkers but substantial numbers of men and women in ‘early mid-life’ also engage in heavy drinking (Emslie *et al*. 2009). The proportion of people aged 25 to 44 years in the UK who report drinking over ‘recommended’ weekly amounts of alcohol (21 units for men, 14 for women) is similar to that among 16 to 24-year-olds (men 26% and 21% respectively; women 19% and 23% respectively) (ONS 2011b). Alcohol-related death rates doubled for both men and women aged 35 to 54 years between 1991 and 2009 (ONS 2011a). A recent systematic review of UK drinking trends over the last 30 years (Smith and Foxcroft 2009) highlighted an increase in drinking in middle and older age groups – particularly among women – and suggested this could be due to alcohol becoming more affordable as well as greater affluence and a dearth of alcohol education aimed at this age group.

Qualitative research on alcohol has also focused on the experiences of young people. Excessive drinking is seen by both young men and women as a routine occurrence so they do not perceive themselves as ‘binge’ or ‘serious’ drinkers (Guise and Gill 2007, Szmigin *et al*. 2008, Seaman and Ikegwuonu 2010). Young adults tend to focus on the behavioural effects of drinking (*e.g*. enough to get drunk, enough to be sick) rather than the number of units of alcohol consumed (Guise and Gill 2007). They participate in moderate, sociable, relaxed drinking as well as the better-documented excessive drinking; the latter is associated with pleasure, escaping from pressure and the generation of ‘drinking stories’ which help to bond friendship groups (Griffin *et al*. 2009, Harnett *et al*. 2000, Szmigin *et al*. 2008). Szmigin and colleagues (2008) argue there is an element of control in excessive drinking among young people which is rarely taken into account and that this ‘calculated hedonism’ is a type of ‘planned letting go which balances out the constrained behaviour they are subject to in the formal structure of everyday life’ (2008: 361). In contrast to this almost exclusive focus on young people, the current paper uses a qualitative approach to explore the social context in which men and women in ‘early mid-life’ drink alcohol, and how constructions of gender influence drinking.

The role alcohol plays in constructions of masculinity has often been commented upon (Campbell 2000). Young men in the UK and the US (de Visser and Smith 2007, Peralta 2007) emphasised the importance of being able to ‘hold’ large amounts of alcohol (usually beer) without throwing up or passing out. They also traded masculine ‘competencies’; sporting prowess could be used to compensate for drinking moderately (de Visser and Smith 2007) while, conversely, competitive drinking could offset a poor sporting performance (Peralta 2007). Men’s hyper-masculine talk during and about drinking often depends on denigrating women and non-hegemonic men (de Visser and Smith 2007, Gough and Edwards 1998, Peralta 2007). However, suggestions that young men dismiss women as not being able to handle alcohol like men (Mullen *et al*. 2007, Peralta 2007) are countered by work in New Zealand (Lyons and Willott 2008) where young men were generally positive about young women who could ‘hold’ their alcohol. Very little qualitative research has studied meanings of drinking among men in mid-life. Robertson’s (2007) work on lay accounts of health among men aged 25 to 40 years found that men described drinking as a kind of ‘release’ from the pressure of paid work and emphasised the importance of escaping daily life through drinking with friends in the ‘masculine’ space of the pub (public house). Harnett (2000) also found that men drank to forget about the stresses of work as well as limiting alcohol consumption because of paid work responsibilities. Although Harnett’s respondents were in their twenties, these findings may be applicable to older men in the labour market.

Until recently, female drinkers were stigmatised, portrayed as victims at risk from predatory men, as sexually promiscuous and/or as lacking in ‘femininity’, and as neglecting their roles as mothers, wives and carers (Day *et al.* 2004, Thom 1997). While men are more likely to drink alcohol than women, to drink excessively and to experience or cause problems related to alcohol, the recent increase in heavy drinking by young British women has led to growing interest in the place of alcohol in women’s lives (Plant 2008). Over the last 50 years, there has been a shift from female patrons being discouraged from entering masculine drinking spaces to women being heavily targeted as consumers (Plant 2008). Alcohol advertising is increasingly targeted at women; for example, young women in the UK identified with the light-hearted image associated with marketing a perry (‘Lambrini girls just wanna have fun’) (Forsyth *et al*. 2007). An Australian study of drinking among female teenagers (Sheehan and Ridge 2001) found that narratives about drinking generally involved laughter and happy memories shared amongst friends. However, their respondents recognised that they were treated differently from their male peers and attributed this to perceptions of young women’s vulnerability. Similarly, work in New Zealand (Lyons and Willott 2008) with respondents in their twenties found that while drunkenness amongst women in their own friendship groups was discussed positively, the same behaviour among ‘outsiders’ was positioned as deviant by both men and women. In particular, older women and women who were very drunk in public were condemned for their drinking and considered embarrassing and ‘slutty’.

In order to develop effective alcohol harm reduction strategies, it is important to understand how men and women in early mid-life themselves perceive drinking and excessive alcohol consumption. This study seeks to fill this gap in the literature by using focus groups to explore the accounts of men and women living in the west of Scotland.

## Methods

In order to explore the social context of drinking in early mid-life (defined here as 35 to 50 years), we conducted focus groups with people who already knew each other and so could draw on existing relationships and shared experiences in the research setting (Kitzinger 1994). Previous work exploring young people’s perceptions of alcohol has used these methods successfully (Lyons and Willott 2008). Ethical approval for the DrAM (Drinking Attitudes in Mid-life) study was granted by the Faculty of Law, Business and Social Sciences Ethics Committee, Glasgow University.

It proved difficult to recruit respondents in this age range. We used a variety of methods including handing out flyers in pubs and on the street, sending email invitations asking people to cascade to friends and colleagues, placing posters on community noticeboards and doctors’ surgeries, phoning community groups and workplaces and advertising on community websites. Once an individual expressed interest, they were asked to recruit a group of up to five people in the same age group who ‘regularly’ drank alcohol. As expected, there was some variation in age in these ‘naturally occurring’ groups; three respondents were younger than 35 and two were over 50 years of age. We also recruited one group of non-drinkers to gain a different perspective on the cultural context of alcohol.

In 2009, 36 respondents (15 men and 21 women) participated in eight focus groups (Table 1). We asked them to complete a short drinking grid to indicate how much they had drunk in the last week, and then converted their replies to units, each of which represents eight grams of pure alcohol which is around the amount of alcohol the average adult can process in an hour. (A small glass of wine contains around 1.5 units of alcohol while a pint of standard lager contains around two units of alcohol). Half of the sample (8 men and 11 women) reported drinking over the ‘recommended’ weekly limit (14 and 21 units for women and men respectively) (Royal College of Psychiatrists 2001). Six of these could be classed as ‘harmful’ drinkers (over 35 units for women and 50 for men respectively) which is associated with a high risk of acute and chronic harm (Department of Health 2007).

**Table 1:** Description of focus groups and participants (N=36)

Focus Group (age range)	Type of group	Mixed or single sex (number)	Alcohol reported in last week	N. drinking at ‘hazardous’ & ‘harmful’ levels^1^	N. with children <18 yrs at home
FG1: Council (44 - 49 yrs)	Work	Mixed sex (n=4)	9-15 units	1 ‘hazardous’	2
FG2: Pub (44 - 50 yrs)	Friendship (met in pub)	Men (n=4)	49-90 units	2 ‘hazardous’ 2 ‘harmful’	1
FG3: Lecturers (34-49 yrs)	Work	Mixed sex (n=4)	21-33 units	4 ‘hazardous’	0
FG4: Friendship (44-48 yrs)	Friendship	Women (n=4)	14-60 units	1 ‘hazardous’ 2 ‘harmful’	3
FG5: Sales (40-50 yrs)	Work	Mixed sex (n=5)	9-36 units	3 ‘hazardous’ 1 ‘harmful’	1
FG6: Community (41-mid 50s?^2^)	Community (deprived area)	Mixed sex (n=7)	0-92 units	0 ‘hazardous’ 1 ‘harmful’	1
FG7: Non-drinkers (25-42 yrs)	Friendship	Mixed sex (n=4)	0 units	N/A	0
FG8: Office (36-47 yrs)	Work	Women (n=4)	0-27 units	2 ‘hazardous’	2

1 Hazardous’ drinking: 22-50 units for men in a week, 15-35 units for women in a week. ‘Harmful’ drinking:>50 units for men in a week, >35 units for women in a week

2 Two of the respondents in FG6 did not give their ages.

All respondents were white and lived in the West of Scotland. This was a socio-economically diverse sample. We used the Carstairs score (Carstairs and Morris 1991) – an area-based measure of social deprivation which takes into account overcrowding, male unemployment, households without a car and low social class – to classify respondents: six respondents lived in affluent areas (Deprivation Category 1 or 2), 12 in deprived areas (Deprivation Category 6 or 7) and the remainder in intermediate areas (Deprivation Categories 3 to 5) (McLoone 2004). Two-thirds (n=24) currently had a partner, and over half (n=20) were parents, although only 10 had children under the age of 18 living with them permanently.

The same researcher (CE) facilitated all the groups. After an explanation of the study and assurances about confidentiality, respondents were asked to give written informed consent and permission for the discussion to be tape-recorded. The topic guide included changes in drinking over time, occasions when respondents had drunk more than they intended, reasons for reducing drinking, and men’s and women’s drinking. Respondents were given £20 gift vouchers.

Focus groups were transcribed verbatim and checked for accuracy. Fieldnotes were written soon afterwards. Transcripts were read repeatedly and coded thematically; QSR Nvivo was used to facilitate analysis. Following McCracken (1988), analysis moved from the particular (a detailed analysis of language in each transcript) to the general (a comparison of patterns across all the transcripts). Hypotheses were formulated, tested against the transcripts and where necessary reformulated in a cyclical process. Below, we present extracts from respondents’ narratives to illustrate each theme and include details of which focus group they participated in and their estimate of what they had drunk in the last week. All names are pseudonyms.

## Dominant discourse: drinking less as one becomes ‘older and wiser’

These respondents in early mid-life felt their drinking had changed since their twenties; they stated that they now drank less – as they were ‘far too old’ to embarrass themselves through excessive drinking – and were more likely to drink at home than at the pub. Youthful drinking was associated with aiming to become drunk quickly (and so choice of drink was influenced by the price and strength of alcohol content), whereas drinking in early mid-life was characterised as being relaxing, sociable and civilised, often accompanied by good food and conversation and by an interest in the taste, rather than the effect, of alcohol (*e.g.* only drinking a particular brand of gin or preferring certain grape varieties of wine). Some respondents declared that, in contrast to their youth, they would not now feel the need to finish a drink they were not enjoying and felt less pressure from others to drink heavily. Craig’s narrative illustrates this discourse of drinking less as one ages, knowing when to stop and being easily able to resist any pressure to drink more. He contrasted his wilder younger self with becoming a ‘wise old owl’ now:

I started drinking about 15 ... cheap and nasty wine (*laughs*)! When I turned 18, I spent my life clubbing in places like Ibiza where it was just drink constantly and every year I went there until I was 30. And then my boy [son] appeared and it was the same as with Debbie, I started to cool it ... I don’t drink nearly as much. I can’t – the older you get, the less you can drink – maybe three or four glasses of wine and that’s me, done, whereas at one time, I could drink three or four bottles of wine ... I think we’ve all done that once, hide the drink – get rid of it some way or another, not leave it [because of peer pressure]. But nowadays, you can be honest and say, ‘I’ve had too much – I’ve had enough, and don’t even say to me have another one, because I’m not interested’. I can do that now. I know when to stop now (Craig, FG1, reported drinking 9 units in last week).

Craig’s suggestion that his drinking had changed when he became a father reflected a common assumption among respondents that alcohol consumption depended on lifecourse position as well as age. A central theme was that as respondents entered mid-life, they had to plan their drinking around their ‘responsibilities’ (particularly children, paid work and driving). Self-control and discipline were necessary in order to prioritise responsibilities and counteract the powerful appeal of alcohol, often referred to using metaphors suggesting the natural force of water (*e.g*. ‘swept along’, ‘go with the flow’). Respondents referred to three demographic stages similar to those discussed by Backett and Davison (1995). First, those who were young with no responsibilities could go straight to the pub from work, drink heavily without eating and then ‘bounce back’ to cope with work the next day or lie in bed for hours to recover at the weekend. Secondly, those who had ‘settled down’ as a couple (but did not have children) often had different priorities and fewer opportunities for socialising due to more demanding jobs, new financial responsibilities and longer commutes. More of their drinking was home based (*e.g*. sharing a bottle of wine over a meal), and drinking during the week (‘drinking on a school night’) was curtailed in order to fulfil work responsibilities, as it was thought to be much more difficult to recover from heavy drinking as one aged. Drinking at weekends was sometimes reduced because leisure time was seen as increasingly ‘precious’. Thirdly, respondents with children were perceived to have many more limits on their drinking. Both fathers and mothers in the sample felt that their drinking had reduced when their children were very young – particularly because of the difficulty of getting up early to look after young children after a heavy night’s drinking – but women tended to introduce the topic and talk about it in more detail, particularly in the two all-female focus groups (FG4 and FG8). For example, Craig (see excerpt above) echoed the point that his female colleague made about reducing her drinking once she had children, but he did not discuss the topic in detail. In contrast, Isobel (mother of three pre-teenage children) described in detail how she and her friends (‘as women’) had a ‘responsible approach’ to alcohol because of childcare responsibilities:

My drinking is controlled by my children. (*Laughing*) ... You’re up early in the morning, packed lunches, hangover, school run – it doesn’t work! ... One of the husbands takes the kids, so that ... the mums can sit down minus children, husband, dishwashers and have a drink and a chat ... We actively get dressed, get changed, hair done, makeup on ... what happens at the start of the evening is we all tell each other what we’re doing in the morning. We actually, as women, discuss who’s got what responsibility ... ‘right, we’re not making it late, what time are we stopping at?’ ... In our group, nearly all the husbands either work away from home or work shifts and are on call. So the women always have to have a responsible approach. They can’t just go out, get really drunk and then worry about not going out in the morning (Isobel, FG8, 0 units).

Some women were very critical of mothers who consumed alcohol while caring for their children. Women discussed how they tried to avoid, or at least limit, their children seeing them under the influence of alcohol and described how they continued to plan their drinking around older children too. For example, respondents in FG4 (all women) had children ranging from 11 to 25 years and discussed how as ‘main carers’ they often monitored their drinking to take account of what their offspring were doing:

And I think as women, you’re the main carers for your child ... you’re still taking all that responsibility on board, so you’re probably much more aware if something happens in the middle of the night, especially when they are younger. And in an emergency, somebody has to be sober ... you’re very aware that you can’t have two glasses of wine, and then, what if something happens? (Stella, FG4, 26 units).

I know ... our son is now at a stage where he’s out on a Saturday night at the under-18s disco and comes back about half ten at night ... and although we’re sitting having a drink, I think it’s because we’re now in a situation where, well I know I could get in a taxi and go and get him, whereas when he was younger, I would maybe not see that as an option. But still aware ... I don’t want to be too well oiled if he’s not in yet (Jenny, FG4, 14 units).

Driving was another ‘responsibility’ which influenced drinking alcohol. Women who lived in the suburbs discussed driving to social occasions in order to save money and for safety reasons, which meant they did not drink alcohol. Both men and women also described ‘taking the car’ as a useful strategy to employ if they did not want to drink. However, most discussions centred on worries about driving the morning after drinking, either to get to work or to transport children to school or leisure activities. Many respondents described how their job depended on having a driving licence and their horror of (being caught) driving over the limit the next morning. Again, this reinforced the idea that drinking in early mid-life needed to be planned rather than spontaneous. Andy described how he planned his drinking around his driving, as he needed his licence to work, while Stella explained how she checked whether she was over the limit before deciding whether to drive the next morning:

I think everyone – certainly our age – has had it pushed into us for a long time and I certainly know people who’ve been breathalysed the next day and lost their licence ... it’s a sobering thought ’cause my job depends on my having a licence (Andy, FG1, 13 units).

We’ve actually got a wee breathalyser in the house ... You maybe get up [the next morning] and take your daughter to choir and you are WAY over and you just can’t, so you send them down in a taxi and it actually makes you stop and think ... But it [drinking] has to be planned, doesn’t it? You can’t just be impulsive in that respect (Stella, FG4, 26 units)

## Challenges to the dominant discourse: really older and wiser?

As the focus groups progressed, different narratives appeared to challenge the idea that respondents drank less as they got ‘older and wiser’. This is perhaps not surprising, given that half of the sample exceeded the ‘recommended’ limits for weekly drinking. Each challenge is discussed in turn.

### Drinking stories and challenges by others

In almost every group, respondents told stories about relatively recent drinking exploits which contradicted previous statements about planned, moderate drinking. These included having the intention of ‘going for one’ but then ‘drinking the night away’ and drinking ‘pranks’ (topping up someone’s glass without them knowing, spiking drinks and ‘scary drunken antics’). It was clear in the heavier drinking groups (FG2, 3, 4, and 5) that these stories contributed to a shared identity:

Some of them [work colleagues] are awfully dull and want to leave early (Bill, FG3, 33 units).

Not in our circle! (*Laughter*) (Julie, FG3, 21 units).

You’ve got it in your mind ‘I’ll have a couple of drinks but I’m not getting really drunk, ’cause I need to get up for work in the morning’. And then you just … (Jody, FG5, 9 units).

Once you’ve had it, you’re like that, ‘Oh, I’ll just have another one. Oh, I’ll just have another wee one’ (*Laughing*) (Grace, FG5, 21 units).

Three and four in the morning, and then you’re like that, ‘Who are we going back to? Have you got any wine?’ (*Laughing*) … It’s like none of us will turn round and say, ‘that’s it, we’ve had enough, let’s go’. It’s like we’re all there to the death and looking for more (*Laughing*) (Rach, FG5, 18 units).

You’re always looking for that one last glass (Pat, FG5, 36 units).

FG5 was unusual in that the respondents were less concerned about presenting an image of themselves as responsible, social drinkers. Their sales jobs required them to work evenings – sometimes promoting alcohol – and they often socialised together after work. Perhaps because of their work culture, they were unusually open about the effects of alcohol (*e.g*. deliberately choosing drinks that would get them drunk faster, blackouts, being asked to leave venues because they were drunk, not knowing where they were or what they were doing).

Apart from respondents in FG5, while women acknowledged that they did sometimes get drunk, they emphasised that this was not the ‘purpose of their evening’. However, men were more direct about their intentions to get drunk although they often emphasised that there was a reason for this decision and that getting drunk would not mean that they neglected their responsibilities:

I’ve seen myself ... coming up the road ‘I’m getting drunk tonight’. But I’d had a right bad week. ‘That’s it. Blow out tonight’ (Gerry, FG3, 29 units).

There will be certain nights you’ll decide, ‘yeah I’m getting drunk and I know it, I don’t care, ’cause I’m enjoying it!’ So you know that OK, I can have a few more, I’m still going to get home safe, I’m not going to disgrace myself, I’m going to suffer in the morning, but I’ll still get up and go to work ’cause I’m enjoying myself right now, I’m enjoying the company, so I’ll take the pleasure and pain (Hugh, FG2, 75 units).

One strength of focus group data is that people can choose whether to agree with or question others’ accounts. Respondents used humour to disagree with other people’s accounts of mid-life drinking. For example, Alison, a 47-year-old mother of two teenagers, explained how she was now happy to leave half a glass of wine when she felt she had drunk enough. Her colleague, Vicky, laughingly disagreed, whispering ‘blasphemy!’ to indicate that she would not do this. Other interjections more directly challenged the veracity of accounts:

I don’t drink because I just cannot justify the cost. If I was in Glasgow every weekend, say Friday and Saturday night with my friends, it would be at least £50, £60 a night? ... I just cannot … (Barbara, FG1, 15 units).

Yeah but that’s what you were doing when I first met you! (Andy, FG1, 13 units).

And then you get the sort of a dizzy kind of a feeling (*Laughing*) ... you’re sitting, you go to get off the bar stool to go to the toilet and you just feel yourself going ... (Jody, FG5, 9 units).

That would stop me, that would stop me from drinking, going that way (Grace, FG5, 21 units).

I’ve seen you that way, don’t give us that! (Rach, FG5, 18 units).

I’ve actually went to dominos and not drunk at all, just drunk juices (Jeff, FG6, 20 units).

Is that a fairy tale?! (George, FG6, 3 units).

### Lifecourse transitions

Descriptions of lifecourse changes also called into question the smooth transition from being young with no responsibilities, to being settled in a couple, to having children, and the suggestion that people gradually drank less as they progressed through these lifecourse stages. Respondents who separated from their partner in their thirties described how they started to go out and drink more. Hugh referred to the breakdown of friendships which accompanied separating from his partner and emphasised the importance of *men* supporting each other in the pub during hard times, while Barbara’s description of her move from non-drinker to ‘party animal’ after her divorce suggested the need (for women) to make up for lost time:

For a lot of men the pub is the place that you socialise.. Late thirties and I didn’t really HAVE a social circle anymore. I split up with my ex and it was like finding your way again. My mate said ‘come and meet some people’. It’s just about –‘OK, that person is quite a good guy ... he can come and join our company, he’s probably a bit worse at the moment’ (Hugh, FG2, 75 units).

I got married young, had my son young ... I was a non-drinker until the age of 39 when I got divorced and hit the social scene ... maybe a bit of a party animal for about five or six years and now it’s calmed down ... I still need to feel as if I can party with the best of them! That might be a kind of common theme in amongst women of a certain age, with no men to hold us back (Barbara, FG1, 15 units).

Like Barbara, women in the study described reducing (or stopping) drinking when their children were young and then drinking more as their children became less reliant. However, the narratives from women in FG4 called this into question. These women were unusual in having dependent children and also being one of the heavier drinking groups. They started off by endorsing the narrative (older people do not NEED to drink) but later in the interview, Jenny and Cath (with two and three children, respectively, under 18 years of age) discussed how the stresses of paid and unpaid work sometimes led to heavier drinking at home:

We’re mature ladies, we’ve all got personalities and we don’t actually NEED a drink for socialising. We ENJOY a drink but we don’t need it to go and talk to folk … (Jenny, FG4, 14 units).

I find it more as a relaxing thing, not something to get your confidence (*agreement from others*) at night if I’m sitting in the house and the kids are away to bed, I think ... nice glass of wine ... and chill (Cath, FG4, 39 units).

[later]

Well, I know for me [I drink more when] I’ve had a CRAP day at work and I’ll come in and ... the first one’s gone before it’s even touched the bottom of the glass, practically! Then you’re onto your second one and maybe by the third one you’ve calmed down (Jenny, FG4, 14 units).

That’s exactly what I was going to say. I can if I’ve had a ... bad day with children, just go [gulping] ... and that’s terrible. I need a drink. I don’t actually NEED it, but I do (*agreement from others*). Go upstairs and sit with a glass of wine. Be fine after that (Cath, FG4, 39 units).

### Pressure to drink

Respondents also told stories which challenged the idea that people in mid-life invariably experience much less pressure to drink from friends, and can easily resist this. They described how their intention not to drink alcohol – or to stop drinking – was sometimes just not accepted and illustrated this through the repeated chants of the group (*e.g*. ‘go on, go on, go, on, just the one’, ‘take one, take one’, ‘just leave the car, just leave the car’ or ‘another one for the road’). Respondents explained that this ‘friendly pressure’ was about everyone being ‘in the same boat’, drinking together and being sociable and relaxed. Alcohol was perceived as a ‘social lubricant’ making the evening more fun and interesting. Respondents described finding it difficult to get into the conversation, feeling like an outsider and being bored by repetitive stories when they went out and did not drink alcohol.

This pressure to drink was illustrated well by respondents who were non-drinkers (FG7). They described how difficult it was for people to understand and accept that they did not drink alcohol; as one woman said, ‘it’s not just socially accepted that people drink – it’s socially EXPECTED’. Most of this group had experience of living in different cities and described receiving a more aggressive reaction from people in the west of Scotland (‘what do you MEAN you don’t drink?’ and ‘well, you’ll have one with me!’). They found themselves constantly having to justify why they did not drink and described being perceived as judgemental or thinking they were superior to others. They also commented on how much interaction in their age group consisted of people talking about going to the pub. They inverted the common cultural portrayal of drinkers as ‘fun’ and non-drinkers as ‘boring’, so that people who did not drink were characterised as entertaining, creative, witty, making real connections with other people and taking responsibility for themselves, while drinkers were portrayed as dull, having repetitive conversations, having shallow relationships with others propped up by alcohol and being irresponsible and unimaginative.

There were gender differences in how respondents described the pressure to drink. Women talked about the problem of monitoring drinking in home settings, where a good hostess would continually top up glasses, and the difficulty of declining a(nother) drink without an ‘acceptable’ reason. Three women in their late forties demonstrated this point:

That’s what I always said to them –‘I’m detoxing’, and everybody was fine. It’s a reason [for not drinking], it’s like you’re saying with diets: if you’re on a diet people accept that, don’t they? But you have to give a reason, if you just say ‘I’m just cutting out alcohol’ then people question it (Stella, FG4, 24 units).

That’s like what I was saying earlier, wasn’t it? People say ‘why?’ (Cath, FG4, 29 units).

Well, they’ve stopped asking us if we’re pregnant, I take it! (*laughter*) (Susan, FG4, 60 units).

Women discussed how they would meet each other for coffee or lunch and not drink alcohol. In contrast, the options for men seemed to be more limited. Respondents in most of the heavier drinking groups (FG2, FG3, FG5) found the idea of men going out for coffee or a meal together, rather than a pint, unlikely or laughable. For example, Graham asserted that he would not see his friends if he did not go to the pub:

I think alcohol ... is a way of life. It’s a cultural thing in Scotland definitely. I know there’s serious issues with it, but we’re all – we all drink to socialise ... If you don’t go to the pub, you’d never see anyone ... Fergus and I hardly ever go for a coffee. Do we? (Graham, FG2, 29 units).

(*Puts on effeminate voice*) Go for a skinny latte (Ewan, FG2, 90 units).

No. I’d probably find that a bit weird ... I mean sometimes I come into the pub if my (football) team’s playing about 12.30 kick off ... and I’m just like, ‘ach, it’s too early, I don’t want a drink yet’. I’m in for the craic [banter] and the game ... You walk over with a glass of coke and it’s just … (Fergus, FG2, 45 units).

Oh! Abuse! (Ewan, FG2, 90 units).

‘Oh, here comes the gay boy’, do you know what I mean? (*laughs*) ... Oh God if you walk over with half a pint of beer, ‘what’s wrong with you, are you skint?’ You know. But I can take it! That’s just our company! (Fergus, FG2, 45 units).

This extract from the all-male focus group (FG2) also demonstrates the insults and jokes which greeted men’s purchase of soft drinks or half pints and called into question their ‘masculinity’. Although some men felt that there might be less peer pressure in smaller groups, or among more educated people, they still commented on the pervasive influence of the ‘macho’ culture in the west of Scotland which involved drinking until ‘falling down drunk’ or being expected to finish a bottle of whisky once it had been opened.

## Discussion

Initially, respondents in this qualitative study of drinking in early mid-life suggested that they only drank alcohol in moderation and instead problematised youthful (binge) drinking. However, these initial assertions that respondents were ‘older and wiser’ were undermined by drinking stories re-told within friendship groups, jokes which questioned stories of responsible drinking, and accounts of continuing peer pressure to drink. Although we did not interview a focus group sample of young people, when we compare our findings with research on younger people there are many parallels (Griffin *et al*. 2009, Szmigin *et al*. 2008); for example, respondents in early mid-life also saw drinking as, first and foremost, an intensely social activity associated with fun, humour and relaxation and incorporated tales of drinking into their group identities. The accounts from the non-drinkers in our study revealed the extent to which (excessive) drinking remains normalised in this age group in Scotland; NOT drinking is the behaviour that requires explanation. Such findings are consistent with research highlighting the pervasiveness of binge and heavy drinking as part of British culture and friendship groups, rendering it normal, routine and enjoyable (van Wersch and Walker 2009).

There were, however, some features that differentiated these accounts from those of younger people. Norms around drinking were heavily influenced by expectations of appropriate behaviour across the lifecourse; for example, drinking to get drunk was seen as much more acceptable for young people who were learning how to drink than for those in mid-life (van Wersch and Walker 2009). Drinking thus could be seen in developmental terms, where people passed through a series of stages from alcohol apprentice to mature and responsible drinker. However, if a stage had been missed out or not fully experienced (*e.g.* early parenthood inhibiting opportunities to drink to get drunk), this could sometimes be revisited at a later date (*e.g.* being a ‘party animal’ when divorced in one’s thirties, drinking excessively as a mature student). While many respondents in early mid-life did (eventually) acknowledge that they sometimes got drunk, they worked hard discursively to make it clear that this was age and stage appropriate (*i.e*. did not result in them being sick or making a fool of themselves). Their accounts suggested a long list of considerations (*e.g*. work responsibilities, hangovers, wasting weekend time, driving, childcare, cost) in order to (be seen to) meet their responsibilities as workers, parents and/or good citizens before they felt able to embark on the ‘controlled loss of control’ described by Szmigin and colleagues (2008). Accounts suggested that this series of considerations ebbed and flowed over the lifecourse according to family and work responsibilities, challenging the notion that people simply decrease their alcohol consumption as they age.

These respondents in early mid-life constructed unrestrained and problem drinking as being younger people’s behaviour. Such problematising of other drinkers is reported elsewhere. For example, heavy-drinking older women (>35 units) positioned younger women drinking heavily in public as problematic (Rolfe *et al*. 2009); younger middle class women positioned women from lower social classes (‘chavvy girls’) as vulgar, uncouth and excessive drinkers (Rúdólfsdóttir and Morgan 2009) while younger adults positioned older people, especially women drinking heavily in public, as embarrassing (Lyons and Willott 2008). This process of ‘othering’ shows how health and health behaviours are used as identity strategies to contrast the healthy, restrained Self with the unhealthy, out of control Other (Crawford 2006). In this way, respondents in our study were able to maintain their identities as mature and responsible drinkers while simultaneously positioning ‘controlled’ (heavy) drinking as acceptable when responsibilities were met. It seems that control is key to the acceptability of drinking in most age and gender groups. Losing self-control is considered unacceptable among female drinkers of any age (Lyons and Willott 2008, Rolfe *et al*. 2009, Rúdólfsdóttir and Morgan 2009) while drinking heavily but tolerating excessive amounts of alcohol whilst maintaining a semblance of control is linked to hegemonic masculinity (Campbell 2000).

In terms of gender, both men and women saw drinking as pleasurable and a fun way to socialise, although women had more concerns about safety, and explicitly expected to take more responsibility for children. Interestingly, both men and women experienced pressure to drink and referred to alcohol both as a reward and a way to recover from – or survive – the rigours of paid work, unpaid work in the case of women, and other stresses. People manage the tensions between, and attempt to balance, ‘control’ and ‘release’ (or ‘denial’ and ‘pleasure’) through a variety of (health) practices (Crawford 2006, Robertson 2007); thus heavy drinking (as a release from tension, an escape from the responsibilities of life, a means of improving mood through laughter with friends) can be seen as part of the process of maintaining good health for both men and women. However, in line with previous research, women provided examples of how they stayed in control of their drinking, and noted explicitly that when they drink they are not intending to get drunk. Despite huge secular changes in gendered patterns of drinking, women still need to undertake discursive work to position themselves as respectable (rather than manly or promiscuous) when drinking heavily (Lyons and Willott 2008, Rolfe *et al*. 2009, Rúdólfsdóttir and Morgan 2009).

In contrast, male participants were able to state that they were going out with the intention of getting drunk (albeit while still demonstrating they could fulfil their responsibilities). Excessive and public consumption of alcohol with other men has been a traditional marker of masculinity in Western cultures (Campbell 2000, Day *et al*. 2004, Gough and Edwards 1998). The pub is especially salient here; a recent large study of heavy drinkers in the UK West Midlands (Orford *et al*. 2009) demonstrated its role as a micro-community which helped maintain relationships. Such findings echo the ways in which men talked about going out to drink with friends in the west of Scotland. It appeared that men had fewer options to socialise apart from the pub. While women would get together with friends at a café, restaurant or people’s homes, men in this study clearly aligned such meeting places as feminine, and used humour and other devices to highlight how meeting male friends in these sorts of places would be demonstrating versions of subordinated masculinities and male hierarchies (e.g. gay men, effeminate men). Not drinking alcohol also led to such responses, as did not drinking in the ‘proper’ way, such as having a half pint. Here hegemonic notions of masculinity were firmly aligned with drinking pints or whisky, drinking heavily, and drinking at the pub, and there seemed surprisingly little room to manoeuvre outside these behaviours.

Our study has a number of limitations. In focus groups, dominant views (whether strongly expressed or articulated by the majority) can close down discussion. It was notable in this study, however, that respondents frequently challenged others’ views. Focus groups can also show how drinking is linked to constructions of individual and group identity. Like other qualitative studies, our respondents were self-selected and it could be argued that our sample was biased toward respondents with fewer home responsibilities who drank heavily. We advertised for people who drank ‘regularly’ but it could be that those who were most interested were heavier drinkers or that people without young children found it easier to attend a focus group. However, respondents who were parents (20 out of 36 respondents) did reflect back to having younger children. There was also a range of drinkers who participated, including non-drinkers (n=4) and ‘moderate’ drinkers (n=13) as well as hazardous or harmful drinkers (n=19). To some extent our approach managed to get beyond self-presentations as responsible drinkers, but there are likely to be presentational aspects due to the presence of the interviewer, and rhetorical constructions that arise as part of the interview context (Radley and Billig 1996).

Cross-sectional surveys usually find that alcohol consumption reduces in older age groups (ONS 2011b), but cannot distinguish between ageing, secular events and characteristics of birth cohorts (Emslie *et al*. 2009). However, a sophisticated analysis of middle-aged and older Danes found the declining probability of heavy drinking by age disappeared after adjusting for period effects and birth cohort; women who were young adults in the 1960s and 1970s had an increased probability of heavy drinking compared to older cohorts (Bjørk *et al*. 2008). Some research suggests moderate drinking in older age may be beneficial (Chen and Hardy 2009), but heavy drinking patterns which remain unchanged after the age of 65 are likely to be detrimental. Thus we now require greater understanding of the factors that influence patterns of drinking behaviours in different age groups.

This study provides a starting point for exploring drinking behaviours among adults in early mid-life. Our findings point to three areas which may be of interest to health promoters, policy makers and other researchers. First, our study demonstrates that heavy drinking is normalised in early ‘mid-life’. Drinking excessively is associated with pleasure and sociability and linked to both gender and group identity construction in mid-life as well as in youth. Thus, health promoters and policy makers need to consider this age group more closely. Secondly, health education messages could build on the social meanings of alcohol consumption we have identified for this population. For example, it may be possible to develop the ‘acceptable’ reasons (such as detoxing or driving) identified by respondents for resisting the ‘friendly pressure’ from peers to have a(nother) drink. Thirdly, more research needs to focus on gender and alcohol in order to identify harm reduction strategies which are acceptable to both men and women. Van Wersch and Walker (2009: 132) point out that ‘binge-drinking is *the* form of “entertainment” and fun for a large group of British people’ and question what alternatives there are for social enjoyment. This question seems particularly pertinent to men, given the lack of opportunity for men to socialise without alcohol identified by respondents in this study.

## References

[b1] Backett KC, Davison C (1995). Lifecourse and lifestyle: the social and cultural location of health behaviours. Social Science and Medicine.

[b2] Bjørk C, Thygesen LC, Vinther-Larsen M, Grønbæk MN (2008). Time trends in heavy drinking among middle-aged and older adults in Denmark. Alcoholism: Clinical and Experimental Research.

[b3] Campbell H (2000). The glass phallus: pub(lic) masculinity and drinking in rural New Zealand. Rural Sociology.

[b4] Carstairs V, Morris R (1991). Deprivation and Health in Scotland.

[b5] Chen L, Hardy C (2009). Alcohol consumption and health status in older adults. Journal of Aging and Health.

[b6] Crawford R (2006). Health as a meaningful social practice. Health (London).

[b7] Day K, Gough B, McFadden M (2004). ‘Warning! Alcohol can seriously damage your feminine health’. A discourse analysis of recent British newspaper coverage of women and drinking. Feminist Media Studies.

[b8] de Visser RO, Smith JA (2007). Alcohol consumption and masculine identity among young men. Psychology and Health.

[b9] Department of Health (2007). http://www.asb.homeoffice.gov.uk/uploadedFiles/Members_site/Documents_and_images/Drinking/AlcoholStrategyJune07_009.pdf.

[b10] Emslie C, Lewars H, Batty GD, Hunt K (2009). Are there gender differences in levels of heavy, binge and problem drinking? Evidence from three generations in the west of Scotland. Public Health.

[b11] Emslie C, Mitchell R (2009). Are there gender differences in the geography of alcohol-related mortality in Scotland? An ecological study. BMC Public Health.

[b12] Forsyth A, Galloway J, Shewan D (2007). Young people’s street drinking behaviour: investigating the influence of marketing and subculture. Alcohol Insight.

[b13] Gough B, Edwards G (1998). The beer talking: four lads, a carry out and the reproduction of masculinities. The Sociological Review.

[b14] Griffin C, Bengry-Howell A, Hackley C, Mistral W, Szmigin I (2009). ‘Every time I do it I absolutely annihilate myself’: loss of (self)-consciousness and loss of memory in young people’s drinking narratives. Sociology.

[b15] Guise JMF, Gill JS (2007). ‘Binge drinking? It’s good, it’s harmless fun’: a discourse analysis of accounts of female undergraduate drinking in Scotland. Health Education Research.

[b16] Harnett R, Thom B, Herring R, Kelly M (2000). Alcohol in transition: toward a model of young men’s drinking styles. Journal of Youth Studies.

[b17] Kitzinger J (1994). The methodology of focus groups: the importance of interaction between research participants. Sociology of Health and Illness.

[b18] Leyland A, Dundas R, McLoone P, Boddy FA (2007). Cause-specific inequalities in mortality in Scotland: two decades of change. a population-based study. BMC Public Health.

[b19] Lyons AC, Willott SA (2008). Alcohol consumption, gender identities and women’s changing social positions. Sex Roles.

[b20] McCracken G (1988). The Long Interview.

[b21] McLoone P (2004). Carstairs Scores for Scottish Postcode Sectors from the 2001 Census.

[b22] Mullen K, Watson J, Swift J, Black D (2007). Young men, masculinity and alcohol. Drugs: Education, Prevention and Policy.

[b23] ONS (2011a). http://www.statistics.gov.uk/statbase/Product.asp?vlnk=14496.

[b24] ONS (2011b). General Household Survey 2007: Smoking and Drinking among Adults, 2009.

[b26] Orford J, Rolfe A, Dalton S, Painter C, Webb H (2009). Pub and community: the views of Birmingham untreated heavy drinkers. Journal of Community and Applied Social Psychology.

[b27] Peralta RL (2007). College alcohol use and the embodiment of hegemonic masculinity among European American men. Sex Roles.

[b28] Plant ML (2008). The role of alcohol in women’s lives: a review of issues and responses. Journal of Substance Use.

[b29] Radley A, Billig M (1996). Accounts of health and illness: dilemmas and representations. Sociology of Health and Illness.

[b30] Robertson S (2007). Understanding Men and Health. Masculinities, Identity and Well-being.

[b31] Rolfe A, Orford J, Dalton S (2009). Women, alcohol and femininity. Journal of Health Psychology.

[b32] Royal College of Psychiatrists (2001). Alcohol – Can the NHS Afford It?.

[b33] Rúdólfsdóttir AG, Morgan P (2009). ‘Alcohol is my friend’: young middle class women discuss their relationship with alcohol. Journal of Community and Applied Social Psychology.

[b34] Seaman P, Ikegwuonu T (2010). http://www.jrf.org.uk/publications/young-people-alcohol-influences-drinking.

[b35] Sheehan M, Ridge D (2001). ‘You become really close … you talk about the silly things you did, and we laugh’: the role of binge drinking in female secondary students’ lives. Substance Use and Misuse.

[b36] Smith L, Foxcroft D (2009). http://www.jrf.org.uk/sites/files/jrf/UK-alcohol-trends-FULL.pdf.

[b37] Szmigin I, Griffin C, Mistral W, Bengry-Howell A, Weale L, Hackley C (2008). Re-framing ‘binge drinking’ as calculated hedonism: empirical evidence from the UK. International Journal of Drug Policy.

[b38] The Scottish Government (2008). Changing Scotland’s relationship with alcohol: a paper on our strategic approach.

[b39] Thom B (1997). Women and alcohol: a policy dilemma. Policy Studies.

[b40] van Wersch A, Walker W (2009). Binge-drinking in Britain as a social and cultural phenomenon: the development of a grounded theoretical model. Journal of Health Psychology.

